# The transition to competency-based pediatric training in the United Arab Emirates

**DOI:** 10.1186/s12909-015-0340-3

**Published:** 2015-04-01

**Authors:** Halah Ibrahim, Hossam Al Tatari, Eric S Holmboe

**Affiliations:** 1 Tawam Hospital, Department of Academic Affairs, 15258, Al Ain Abu Dhabi, United Arab Emirates; 2 Johns Hopkins Graduate School of Education, Baltimore, MD USA; 3 Tawam Hospital, Department of Pediatrics, Al Ain Abu Dhabi, United Arab Emirates; 4 Accreditation Council for Graduate Medical Education, Chicago, Ill USA

**Keywords:** Pediatrics, Competency-based medical education, Outcomes-based training, International medical education, Residency

## Abstract

Although competency-based medical education has become the standard for physician training in the West, many developing countries have not yet adopted competency-based training. In 2009 in the United Arab Emirates, the government regulatory and operational authorities for healthcare in Abu Dhabi mandated a wide-scale reform of the emirate’s postgraduate residency programs to the competency-based framework of the newly formed Accreditation Council for Graduate Medical Education-International (ACGME-I). This article briefly describes the rationale for competency-based medical education and provides an overview of the transition from traditional, time-based residency training to competency-based postgraduate medical education for the Pediatrics residency programs in Abu Dhabi. We will provide data on the initial impact of this transition on resident performance and patient outcomes in a Pediatrics residency program in an academic medical center in the United Arab Emirates.

## Correspondence article

In the century since Halstead and Osler redefined postgraduate training in the United States, countries throughout the world have reformed their medical education systems to be more responsive to the changing needs of society. As the complexity of the healthcare system increases, so does the need for competent physicians. Regulatory bodies worldwide, including the Accreditation Council for Graduate Medical Education (ACGME) in the United States, the General Medical Council (GMC) in the United Kingdom, and the Royal College of Physicians and Surgeons of Canada (RCPSC), have responded by developing national educational change initiatives grounded in competency-based medical education (CBME). With the 21^st^ century globalization of medicine, these Western-developed frameworks have significantly influenced postgraduate medical training in the East. The CanMEDS model has been adopted extensively around the world [1]. More recently, several countries, including Singapore, the United Arab Emirates (UAE) and Qatar, have restructured their residency training programs around the core competencies of the US-based Accreditation Council for Graduate Medical Education- International (ACGME-I). Although academic scholars continue to debate over the educational and clinical outcomes of competency-based education, it has become the worldwide standard for postgraduate training of physicians. By aligning the requisite competencies desired in health professional trainees with each country’s health care priorities, competency-based training directly integrates graduate medical education (GME) with the health and healthcare needs of populations [2,3].

Outcomes-based learning was first introduced as an instructional strategy by behavioral psychologists as early as the 1950′s [4], but it was not uniformly adopted until decades later when several influential reports called for a transition to competency-based training and regulatory bodies such as the ACGME and the RCPSC mandated CBME as a requirement for accreditation [5,6]. There are multiple driving forces for this widespread adoption of CBME. As an instructional strategy, competency based training is based on sound pedagogy which promotes learner-centeredness [7]. CBME also provides a framework for curricula and assessments, as well as flexibility and an opportunity for self-directed learning [6]. The recently published Flexner centenary report suggests implementing defined outcomes and identifying minimum competencies for graduates as a means to promote transparency and accountability of the medical education system and alleviate patient safety concerns [8]. These benefits are particularly important in countries with developing healthcare systems, who are actively building a health professional workforce.

This article presents an overview of the educational restructuring and describes the key elements of the transition to competency-based training in the Pediatrics residency training programs in Abu Dhabi, United Arab Emirates from 2010–2013. While we cannot definitely determine cause and effect, we will also provide data on the initial impact of this transition on resident performance and patient outcomes in a Pediatrics residency program in an academic medical center in the UAE as an early case study. Our hope is that the lessons learned from our experience will provide guidance for GME restructuring in other international training programs.

In Abu Dhabi, postgraduate training in Pediatrics is offered in four of the five government teaching hospitals. Without central oversight, standardized curricula and assessments, or guidelines for promotion, inconsistencies developed in the quality of training and in the competence of graduates. Every program faced issues with resident recruitment and retention, and Arab Medical Board pass rates were consistently low. In a country with an ever increasing annual birth rate, the quantity and quality of graduating pediatricians became an issue of national concern. As such, pediatrics was one of the first programs mandated to reform.

To accomplish such a wide-scale educational transformation, an advisory committee consisting of program directors, department chairs, hospital administrators and residents was formed. Based on recommendations from this advisory committee, we focused our efforts in six specific areas that we will describe in greater detail.

1. Invest in faculty development
2. Assessment as an opportunity for growth and development
3. Teamwork
4. Increase focus on ambulatory training
5. Encourage collaboration
6. Address the continuum of medical education

### Invest in faculty development

Faculty engagement and support are critical for successful educational reform. Recent evidence suggests that trainee development, incorporation of formative feedback, and professionalism are all enhanced when the learners have longitudinal, meaningful relationships with their faculty and feel that the faculty are invested in their professional growth [9–11]. As such, in addition to traditional teaching, we incorporated supervision and mentoring of trainees as explicit faculty responsibilities. In order to sustain our efforts, the willingness and ability to educate trainees is now a consideration in the recruitment of all new clinicians. Teaching has also become a component of the job descriptions and appraisals of all Pediatricians in our teaching hospitals. Resident evaluations are an important consideration in all faculty promotion decisions.

### Assessment as an opportunity for growth and improvement

Since CBME emphasizes the *outcomes* of education rather than the *process* of education, assessment becomes a critical component. Through personalized, longitudinal oversight of each trainee, assessment can determine whether or not students have achieved the benchmarked levels of competence. Direct observation and workplace based assessment were emphasized and multisource feedback was performed to provide meaningful formative feedback and an opportunity for self-assessment and professional growth [12]. A significant benefit of competency-based training is that it can identify learner deficiencies in a timely manner and facilitate early remediation [13]. Several of our programs have identified and successfully remediated residents who would have likely continued to fall behind their peers in the previous educational system.

### Teamwork

The current practice of medicine requires physicians to manage patients within a healthcare delivery system and as part of a multidisciplinary healthcare team. This lies in stark contrast to the autonomy of the previous apprenticeship model of training to which our residents were accustomed. We developed interdisciplinary teams based on graded resident responsibility to manage all general pediatric inpatients. Weekly multidisciplinary rounds were established to address all aspects of patient care. This also provided an opportunity to identify and address system errors. Residents have become valuable members of the hospitals’ quality departments and patient safety teams. Issues, such as ambulatory clinic wait times and patient vaccination compliance, have been successfully addressed through resident-led quality improvement projects.

### Increase focus on ambulatory training

Graduate medical education in the UAE has historically focused on acute illness and exacerbations of chronic disease. The shift to chronic disease management and prevention required an increased emphasis on ambulatory training. As a result, we added both a didactic and clinical focus on outpatient medicine in the curriculum. The literature suggests that resident clinics can be understaffed and dysfunctional and have the unexpected side effect of discouraging residents from careers in primary care, which can have negative implications on a nation’s healthcare workforce [14,15]. As such, it was critical to develop well-organized clinics and provide high quality ambulatory training that emphasizes cost-effective, patient-centered outpatient care. Residents were protected during their clinic sessions and coverage was provided to answer the needs of their inpatients. Clinic preceptors were also expected to discuss not only differential diagnoses and effectiveness of treatment, but also health maintenance, prevention and anticipatory guidance.

### Encourage collaboration

International training programs, especially those in developing regions, may lack the resources and expertise to train physicians in all domains, thereby, emphasizing the need to break down silos and develop collaborative training programs. In 2010, a Lancet Commission on education of health professions for the 21^st^ century [16] put forward a vision that “*all health professionals in all countries should be educated to mobilize knowledge and to engage in critical reasoning and ethical conduct so that they are competent to participate in patient-centered health systems as members of locally responsive and globally connected teams.*” Healthcare in the UAE already has a long history of international collaborations, including relationships with the Imperial College of London, Johns Hopkins International and the Cleveland Clinic. We also participated in international educational cooperations and global exchange programs for our students and teachers. It is our hope that UAE teaching programs will, in turn, share resources and partnerships with less developed medical education systems, both within and outside the Arab world.

### Address the continuum of medical education

Medical training is a complex system and it is difficult to reform one level without necessitating change in other domains. In the UAE, undergraduate medical education continues to be comprised of long hours in the classroom and frequent written examinations, but limited hands-on training. The clinical clerkship in Pediatrics provided a critical opportunity to involve students in the active supervised care of the pediatric patient and for introducing students to unfamiliar concepts, such as systems based practice and quality improvement principles, thereby providing the medical school graduates with the requisite competencies for entry into residency training.

## Initial experience at one hospital

Although the impetus for reforming postgraduate training was to develop a competent healthcare workforce that will ultimately improve patient care in the UAE, the question remained whether educational reform to competency-based training actually improves outcomes and is not associated with unintended consequences. To explore these questions, we will provide a case study of the impact of this reform on the oldest and largest Pediatric residency program in the UAE. The Pediatrics residency program at Tawam Hospital was started in 1996. By 2010, the program was near collapse. Board pass rates on the Arab Medical Board examinations were consistently below the national average, residents were transferring to other disciplines, and two recruitment seasons passed without a single applicant to the program. With training reform based on CBME, coinciding with the institution of a national residency match program, improvements were seen in resident recruitment and educational outcomes. In contrast to previous years without applicants, in the 2013 residency recruitment season 12 positions were matched from a total of 196 applicants to the program. In addition, on the Pediatric Arab Board examination, an exam with historically low pass rates, Tawam boasted a 92% pass rate in 2011 (13/14), 90% pass in 2012 (9/10), and 100% pass rate in 2013 (15/15). Studies have demonstrated positive predictive relationships between scores on qualifying examinations and patient care outcomes [17,18].

Improvements in patient care outcomes in the Pediatric Department were also witnessed within this three year time period. As a Joint Commission International-accredited institution, the hospital’s Quality Department meticulously collects clinical outcomes data, including adverse and sentinel events, medication errors and morbidity and mortality reports. Additionally, in mid-2009 Tawam implemented the Patient Safety Net (PSN), a web-based event-reporting system, which serves as a data collection tool for all adverse events, errors, service complaints and near misses in both the inpatient and ambulatory setting. A retrospective review of the hospital’s performance data collected by the Quality Department for the Department of Pediatrics and all Pediatric PSN reports from 2009–2013 was conducted to assess potential changes in patient care outcomes. The study was approved by the Al Ain Medical District Research Ethics Committee (12/71). Despite increases in the number and acuity of patient admissions and in the reporting of adverse events, the number of reported clinically significant adverse events in the Department of Pediatrics decreased significantly (Table 1). Also, the average length of stay for inpatient general pediatric patients decreased from 3.2 days in 2010 to 2.8 days in 2012 and 2013 (Table 2). Faculty and trainee focus on the competencies of professionalism, communication skills, practice-based learning and systems-based practice created an emphasis on patient-centered care and quality improvement that likely contributed to the improved patient outcomes noted. Although it is not possible to prove causation between complex educational interventions and patient care outcomes in the real-world setting, the trends toward improvements in patient care coinciding with increases in the resident complement and service provided, are quite reassuring and do not suggest adverse unintended consequences for patients as a result of the CBME system. It is quite likely that our residents are providing better patient care. More importantly, the experience of providing better patient care as part of their medical training will undoubtedly have a positive impact on the care our residents will provide as future independent practitioners [19].

**Table 1:** Pediatric department adverse events by PSN* reports

	2010	2011	2012	2013	
**Admissions**	2999	3883	4057	4839	
Total PSN reports*	152	170	189	277	p = 0.02
Harm score E & F**	33	29	12	0	p < 0.0001
Percentage***	22%	17%	6%	0%	

*Adverse events reported in hospital’s Patient Safety Network for inpatient Pediatrics and verified by Quality Department. Statistics are calculated independently of residency leadership involvement. Data may be limited by reporting bias whereby hospital employees report less serious events, but are reluctant to report more serious adverse events.

**E-The individual experienced temporary harm and required treatment or intervention; F- The individual experienced temporary harm and required initial or prolonged hospitalization. There were no reported events with more serious harm scores during this time period.

***Percentage is calculated by # events with harm score E or above/total # adverse events.

**Table 2:** **Pediatric department inpatient statistics**

	2010	2011	2012	2013
**Admissions**	2999	3883	4057	4839
**Length of stay**	3.2	2.9	2.8	2.8
**Case mix index**	0.88	1.31	1.35	1.43

Increased case mix index corresponds to increase in diversity and clinical complexity of patients.

## Conclusion

The goal of graduate medical education is to develop competent physicians who can meet the public’s healthcare needs. With changes in technology, economics and population demographics, society’s needs are continuously changing. Accordingly, medical education must continually evolve. Over the past few years, Pediatrics training programs in Abu Dhabi have embraced competency-based medical education in an effort to provide better healthcare for UAE children and their families. All Pediatric residency programs in Abu Dhabi have recently received accreditation by the Accreditation Council for Graduate Medical Education- International (ACGME-I). Early outcome data from the largest Pediatric program in Abu Dhabi are positive in terms of both educational and patient care outcomes. Longitudinal and multi-institutional data are needed. Also, substantial funding is necessary to sustain high quality medical education. Long term sustainability and generalizability to other emerging healthcare systems will require better strategies for funding and innovative ways to improve education without escalating costs. Finally, it has been acknowledged that functional and competent healthcare systems are necessary for the training of competent physicians [20]. It is, thereby, critical that the graduates of our residency programs contribute to the competency and quality of the UAE healthcare and educational system.
